# Novel 3D‐Printed Biophotonic Scaffold Displaying Luminescence under Near‐Infrared Light for Photopharmacological Activation and Biological Signaling Compound Release

**DOI:** 10.1002/adhm.202502163

**Published:** 2025-08-18

**Authors:** Sonya Ghanavati, Ekin Opar, Virginia Alessandra Gobbo, Carlo Matera, Fabio Riefolo, Rossella Castagna, Julien Colombelli, Andrew Draganski, Joshua Baggott, Mika Lastusaari, Pau Gorostiza, Laeticia Petit, Jonathan Massera

**Affiliations:** ^1^ Faculty of Medicine and Health Technology Tampere University Korkeakoulunkatu 3 Tampere 33720 Finland; ^2^ Institute for Bioengineering of Catalonia (IBEC) The Barcelona Institute for Science and Technology (BIST) Carrer de Baldiri Reixac 10 Barcelona 08028 Spain; ^3^ Doctorate Program in Engineering and Applied Science Universitat de Barcelona Barcelona Spain; ^4^ Network Biomedical Research Center in Biomaterials, Bioengineering, and Nanomedicine (CIBER‐bbn) Barcelona 08028 Spain; ^5^ Department of Pharmaceutical Sciences University of Milan Via L. Mangiagalli 25 Milan 20133 Italy; ^6^ Teamit Institute, Partnerships, Barcelona Health Hub Barcelona 08025 Spain; ^7^ Dipartimento di Chimica Materiali e Ingegneria Chimica “G. Natta” Politecnico di Milano Piazza Leonardo da Vinci 32 Milano 20133 Italy; ^8^ Department of Biotechnology Latvian Institute of Organic Synthesis Aizkraukles 21 Riga LV‐1006 Latvia; ^9^ Institute for Research in Biomedicine (IRB Barcelona) The Barcelona Institute for Science and Technology (BIST) c. Baldiri Reixac, 10 Barcelona 08028 Spain; ^10^ Zylö Therapeutics 105A Ben Hamby Dr Greenville SC 29615 USA; ^11^ Catalan Institution for Research and Advanced Studies (ICREA) Barcelona 08010 Spain; ^12^ Photonics Laboratory Tampere University Korkeakoulunkatu 3 Tampere 33720 Finland; ^13^ Department of Chemistry University of Turku Turku FI‐20014 Finland

**Keywords:** drug targeting, implants, luminescence, nitric oxide, optopharmacology, photopharmacology, phototherapeutic window, upconversion

## Abstract

Despite significant efforts in developing novel biomaterials to regenerate tissue, only a few of them have successfully reached clinical use. It has become clear that the next generation of biomaterials must be multifunctional. Smart biomaterials can respond to environmental or external stimuli, interact in a spatial‐temporal manner, and trigger specific tissue/organism responses. In this study, the fabrication of novel 3D‐printed and bioresorbable scaffolds, with embedded crystals that can convert near‐infrared (NIR) light into visible light, is presented. It is demonstrated that these biophotonic scaffolds are not only bioactive and bioresorbable, but can also be promising as a platform for the controlled release or activation of photoactivated drugs locally and on demand, under illumination. The scaffolds are analyzed based on their up‐conversion spectroscopic properties and their chemical stability in simulated body fluid. Furthermore, it is demonstrated that the up‐conversion properties of the scaffolds are sufficient to release the signaling molecule nitric oxide (NO) and to photoisomerize the muscarinic ligand Phthalimide‐Azo‐Iperoxo (PAI), in a controlled manner, upon NIR light stimulus. Finally, to assess their biocompatibility for potential implantation, a preliminary study is conducted with human adipose stem cells cultured in contact with scaffolds. Live/dead assays, morphological analysis, CyQUANT analysis, and ion release measurements confirm that, despite some release of the upconverter crystals, the biophotonic materia and its dissolution by‐products, are biocompatible. These findings highlight the potential of these bioresorbable biophotonic scaffolds for localized drug release in response to NIR light stimuli.

## Graphical abstract

Despite significant efforts in developing novel biomaterials to regenerate tissue, only a few of them have successfully reached clinical use. It has become clear that the next generation of biomaterials must be multifunctional. Smart biomaterials can respond to environmental or external stimuli, interact in a spatial‐temporal manner, and trigger specific tissue/organism responses. Here the feasibility of producing 3D printed scaffolds, loaded with up‐converter crystals enabling spatial and temporal release/activation of drugs, under near‐infrared excitation is demonstrated.

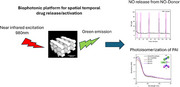

## 1 Introduction

Bioactive glasses (BAG) are biocompatible materials that possess the ability to bond with bone as well as soft tissues, making them highly promising for bone tissue regeneration applications.^[^ ^1^ ^]^ Among bioactive glasses, silicate‐based compositions such as 45S5 and S53P4 have been used in clinical settings for decades.^[^ ^1^, ^2^, ^3^ ^]^ These materials are primarily applied in the form of granules or putty. However, their significant tendency to crystallize during heat processing makes it challenging to shape them into 3D structures without adverse crystallization, which can compromise their bioactivity.^[^ ^4^, ^5^ ^]^ Crystallization has been shown to reduce the ability of bioactive glasses to form a hydroxyapatite (HA) layer upon immersion in body fluids, which is crucial for successful bone bonding.^[^ ^6^ ^]^ Despite the unique interaction of these materials with bone, the possibility of producing an optimal bone graft remains a concern. Several challenges must be addressed in developing effective BAG scaffolds as bone implants, including maintaining the bioactivity of the material, preventing crystallization during sintering, and achieving a porous structure with controlled pore size and interconnected porosity. The scaffolds must mimic the natural structure of cancellous bone, facilitating bone reconstruction in three dimensions.^[^ ^7^ ^]^ Most techniques to process scaffolds rely on glass particles sintering. However, glass devitrification is a common phenomenon occurring upon heat treatment, which, in the case of bioactive glasses, decreases or even suppresses bioactivity.^[^ ^8^, ^9^ ^]^

While traditional bioactive glasses (45S5 and S53P4) cannot be sintered without adverse crystallization,^[^ ^3^ ^]^ modifying the glass composition can help in controlling the crystallization kinetics of BAG. The glass 13‐93, developed by Brink et al.^[^ ^10^ ^]^, is a good example of a silicate glass demonstrating bioactivity (i.e., the ability to precipitate HA upon immersion in an aqueous solution) while possessing a broad hot forming domain.^[^ ^10^, ^11^ ^]^ However, 13‐93 glass showed a lower dissolution rate compared to the traditional bioactive glasses.^[^ ^1^ ^]^ In order to develop glass compositions showing controlled dissolution with a wide hot forming domain, borosilicate glasses have been developed. Borosilicate glasses have also been found to convert more rapidly and more completely into HA, compared to their silicate counterpart.^[^ ^11^ ^]^ Borosilicate glasses also promote angiogenesis and support cell proliferation and differentiation.

Despite the effort from the scientific community in developing new bioceramics for bone reconstruction, it is apparent that no new bioactive glass composition has yet reached the market. This might be due to the minimal improvement in clinical outcome provided by the newly studied compositions. It is unlikely that a new composition will find space in the clinic if the benefit to the surgeon is limited. Therefore, it is crucial to develop multifunctional biomaterials that not only promote tissue regeneration but also act as a platform enabling the controlled release of active agents (pain killers, antimicrobial or anticancer agents, etc). Beyond the slow‐release implantable materials that have been recently approved, like dental antimicrobials and contraceptives,^[^ ^12^, ^13^, ^14^ ^]^ controlled release requires an external input signal, such as light, temperature, or pH changes, to trigger the process.^[^ ^15^, ^16^, ^17^ ^]^ Among others, light is a very convenient stimulus because it is simple to apply using a great diversity of clinically approved devices and can be controlled spatiotemporally. For example, light can control drug release and regulate protein activity, enabling precise modulation of therapeutic effects. In phototherapy and photobiomodulation, light is used to enhance cellular responses, promote tissue regeneration, and reduce pain or inflammation by targeting specific biological processes.^[^ ^18^, ^19^, ^20^ ^]^

To couple illumination to pharmacological action, in recent years, several photoswitchable and photo‐releasable drugs have been developed, such as Phototrexate,^[^ ^21^ ^]^ Phthalimide‐Azo‐Iperoxo (PAI),^[^ ^22^ ^]^ and Nitric Oxide (NO)‐releasing materials. Phototrexate is a photoswitchable analogue of methotrexate, designed to enable light‐controlled inhibition of dihydrofolate reductase (DHFR), a key enzyme in folate metabolism, and developed as a proof‐of‐concept for photoactivated chemotherapy. Structurally, phototrexate incorporates an azobenzene photoswitch through an azologization strategy, allowing it to isomerize between *trans* and *cis* states. In its *trans* form, it exhibits minimal inhibitory activity on DHFR. Upon UV illumination at 375 nm, it undergoes photoisomerization to its cis form, which closely mimics methotrexate and binds DHFR with high affinity, leading to strong enzyme inhibition. The effect can be reversed by exposure to blue light (460 nm) or spontaneous thermal relaxation in the dark. In vitro studies demonstrated that *cis*‐phototrexate effectively reduces cell proliferation, comparable to methotrexate, while the *trans* form remains largely inactive, thus potentially minimizing systemic toxicity. In a zebrafish model, photoactivation of phototrexate resulted in developmental abnormalities and increased mortality, highlighting its potential for targeted chemotherapy. PAI is a synthetic compound designed to modulate the M_2_ muscarinic acetylcholine receptor (mAChR), a receptor implicated in the regulation of heart rate and central nervous system functions, among other functions. In its dark‐adapted (*trans*) form, PAI activates M_2_ muscarinic acetylcholine receptors (mAChRs), with the ability to reversibly toggle its activity upon exposure to UV and blue light.^[^ ^22^ ^]^ PAI consists of a photoisomerizable azobenzene‐based spacer, an allosteric fragment, and an orthosteric Iperoxo‐like moiety. Nitric oxide (NO) is another active agent of interest as it is a crucial bioregulatory molecule with significant physiological roles, including vasodilation and neurotransmission.^[^ ^22^, ^23^ ^]^ Its versatile biological activity makes NO an attractive candidate for therapeutic applications, including wound healing, tissue regeneration, and infection control. S‐nitroso‐N‐acetyl‐D‐penicillamine (SNAP), a compound frequently used for NO release, has been extensively studied for its potential antimicrobial properties.^[^ ^22^, ^23^ ^]^

While controlled NO release using light‐emitting diodes (LEDs) at 460 nm has shown promise, its therapeutic penetration is limited due to tissue attenuation, restricting its application in deeper tissues.^[^ ^24^, ^25^ ^]^ To address this challenge, upconverter phosphors, which convert near‐infrared (NIR) light into visible light, have emerged as a solution for enabling deeper, more controlled phototriggered NO release. In our previous work,^[^ ^26^ ^]^ we demonstrated that 3D biophotonic scaffolds, prepared by the porogen burn‐off technique, that integrate CaWO_4_ co‐doped with Yb^3^⁺ and Er^3+^ crystals into bioactive borosilicate glass, efficiently convert NIR to green emission, leading to the release of NO from SNAP, overcoming the limitations of previous light‐based NO delivery systems.

As opposed to borosilicate glass, phosphate glass composition can be tailored so that the glasses can dissolve congruently, allowing degradation rates to be precisely tuned from weeks to years.^[^ ^27^ ^]^ For example, metaphosphate glasses show good sintering ability, but their fast degradation rate may harm cells and reduce their viability.^[^ ^28^ ^]^ On the contrary, inverted phosphate glasses have been found to degrade more slowly and to be more appropriate when interacting with cells.^[^ ^29^ ^]^ Borosilicate glass in aqueous solution led to the formation of a silica‐rich layer and the formation of hydroxyapatite over time, which might hinder the upconversion process and reduce the drug efficiency. Use of phosphate glass eliminates the concerns associated with high boron content in borosilicate glass, which has been reported to negatively affect cell proliferation.^[^ ^30^ ^]^

The primary objective of this study is to explore the potential of advanced multi‐functional 3D‐printed phosphate glass scaffolds, into which CaWO_4_ crystals are embedded, for photo‐controlled drug delivery using tissue‐penetrating NIR light. A new phosphate glass was developed with the composition in (mol%) 45P_2_O_5_‐ 2.5B_2_O_3_‐ 2.5SiO_2_‐ 10Na_2_O‐ 20CaO‐ 10SrO‐ 10MgO (in %mol) as in Ref. [31]. This composition shows promising results in terms of hot forming domain with a congruent dissolution and, thus, controlled ion release. We aim to develop scaffolds that not only provide structural support but also actively regulate drug release kinetics and modulate cellular responses. In this article, we present a comprehensive investigation of the fabrication and characterization of these innovative implants. Subsequently, we demonstrate the effective release of NO and the controlled drug delivery using PAI as a photoactivated drug model using external NIR 980 nm light. Additionally, we demonstrate that the materials are cytocompatible by cultivating human adipose stem cells in direct contact with the biophotonic scaffold.

## 2 Experimental Section

### 2.1 Scaffolds

#### 2.1.1 Glass and Upconversion Crystal Preparation

Phosphate glass with the composition 45P_2_O_5_‐ 2.5B_2_O_3_‐ 2.5SiO_2_‐ 10Na_2_O‐ 20CaO‐ 10SrO‐ 10MgO in mol% (labeled as X10) was successfully prepared using the melt quenching technique as in Ref. [18].30 g of glass was melted in a platinum crucible, heated at 10 C min^−1^ to 1000 °C, and kept for 30 min at the melting temperature. The glass was cast and annealed at 425 °C for 6 h to release internal stress. The glass was then crushed into powder and ball milled (PULVERISETTE 7, FRITSCH) and sieved to a particle size of <38µm.

The upconverter (UC) crystals, CaWO_4_ crystals, were prepared by solid‐state reaction following the procedure described in Ref. [26]. The synthesis involved heating CaCO_3_ (Alfa‐Aesar, technical grade), WO_3_ (Honeywell‐Fluka, 99%), Yb_2_O_3_ (Sigma–Aldrich, 99.9%), Er_2_O_3_ (Sigma–Aldrich, 99.9%), and Na_2_CO_3_ (Sigma–Aldrich, 99.9%) at 1200 °C in air for 4 h. The crystals were co‐doped with 15 at% Yb^3^⁺ and 0.75 at% Er^3^⁺, with Na⁺ used as a charge compensator to replace Ca^2^⁺, as detailed in Ref. [26]. The resulting particles exhibited various shapes and sizes (≈10–30 µm), consistent with our previous findings.^[^ ^26^ ^]^

#### 2.1.2 3D Printing of Scaffolds

The ink, used to 3D print scaffolds, was prepared by first mixing Pluronic F‐127 (Sigma–Aldrich) in distilled water (25 wt.%). The container was placed in an ice bath, and the solution was stirred for a minimum of 6 h (until it became transparent). As demonstrated earlier,^[^ ^26^ ^]^ incorporating 10 wt.% of the UC crystals into the scaffold matrix (labelled X10UC) was determined to be optimal for enhancing up‐conversion efficiency while maintaining the scaffold mechanical integrity. This balance ensures effective NIR‐triggered photophysical responses. Thus, the glass particles and the UC crystals were mixed with the Pluronic solution at a ratio of 30/70 vol%. The obtained ink was mixed for 20 s and then stabilized in an ice bath for 30 s. A Vibrofix VF1 electrical shaker (IKA‐Labortechnic, Staufen, Germany), at 2500 rpm, was used to homogenously mix the compounds while preventing any bubble formation. The ink was then transferred to a cartridge (Nordson EFD, Bedfordshire, England) and kept at room temperature for 2 h to stabilize before printing. The cartridge was manually attached to the 3D printer (3Dn‐Tabletop, nScrypt, Orlando, USA). SmoothFlow Tapered Dispense (0.41mm, Nordson EFD Optimum SmoothFlow, Westlake, OH, USA) was used. The material feed was pressure‐controlled (13.0–21.0 psi) during the printing process to ensure consistent ink flow. The scaffolds were 3D printed by the robocasting method (also known as direct ink writing) by extruding ink through a nozzle, layer by layer, forming a cylindrical shape with a diameter of ≈3.5 mm and a height of ≈3.3 mm. Scaffolds were finally dried at room temperature (≈21–22 °C) before being sintered at 525 °C for 1h as described in Ref. [31]. Crystals free scaffolds (labelled at X10) were 3D printed using the same procedure. The porosity of the scaffolds was estimated by weighing the scaffolds and measuring the exact diameter and height. A glass density of 2.78 g cm^−3^ was used for the calculation and extracted from Ref. [31].

For the upconversion intensity measurements, excitation was provided by an MDL III 980 2W EL20057 NIR laser operating at 980 nm and 4.2 Wcm^−2^ power density. Upconverted irradiance was collected at a 20 mm distance from the sample surface with a DeltaOHM LP 471 RAD Probe Irradiance Meter (400–1050 nm) connected to a DeltaOHM HD2102.1 Photo/Radiometer. A glass filter (Standa S3S21) was placed between the sample and the detector to block stray laser reflections.

#### 2.1.3 Scaffolds Covered with SNAP to Control NO Release

S‐Nitroso N‐acetyl Penicillamine (SNAP) solution was prepared by dissolving 50 mg of N‐Acetyl Penicillamine ((S)‐2‐acetamido‐3‐mercapto‐3‐methylbutanoic acid, BLDPharm, Catalog no. BD142945; CAS# 15537‐71‐0; Purity: >98%) in 1 mL of dimethyl sulfoxide (DMSO). Then, 100 µL of 1 m sodium nitrite and 100 µL of 1 m hydrochloric acid were added to the solution. The mixture was vortexed thoroughly and incubated at room temperature for 1 h. The formation of the nitrosation product (SNAP) was confirmed by the green color of the solution. The complete conversion of penicillamine to SNAP was confirmed using UV–vis absorption spectroscopy, which showed a peak corresponding to an extinction coefficient of 0.7 mg·mL^−1^·cm^−1^.

Cylindrical‐shaped scaffolds with dimensions of 3.5 mm in diameter and 3.3 mm in height were placed on clean glass surfaces. A total of 50 µL of the SNAP solution was applied to the dry scaffold, which absorbed the solution quickly. The scaffold was flipped using tweezers, and another 50 µL of the SNAP solution was added to the opposite side to cover all the entire scaffold. To promote solvent evaporation, a small portable fan heater was positioned several centimeters in front of the scaffold. The scaffold was shielded from light to prevent the premature release of NO. After an initial drying period of 10 min, an additional 100 µL of the SNAP solution was applied. This process was repeated until 500 µL of the SNAP solution had been applied, resulting in scaffolds with a dark green color. The scaffolds were then examined under brightfield microscopy (10x objective) to confirm uniform deposition of SNAP across all scaffold surfaces. To confirm photoactivation of NO release, the scaffolds were placed at the entry port of a Nitric Oxide Analyzer (Eco Physics, CLD 60). A 980 nm, 0.5 W laser (MIL‐III‐980‐500mW) was positioned ≈12.7 cm from the scaffold. The laser, operating at 980 nm, was turned on and off over a period of 180 min to demonstrate that NO release is photoactivated by the incident light.

#### 2.1.4 Photoisomerization of the Photoswitchable Antiproliferative Compound Phototrexate

**Figure 1:**
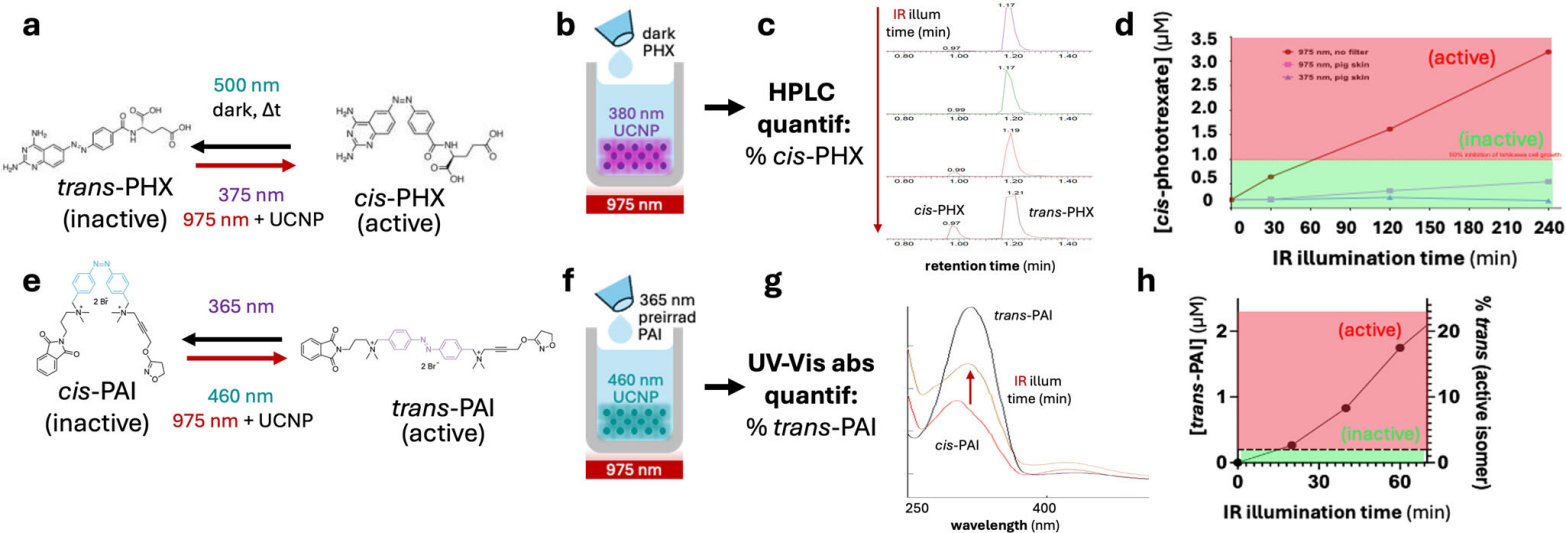
Proof of concept of upconverted photopharmacology using the photoswitchable antiproliferative Phototrexate (PHX) and the photoswitchable muscarinic modulator Phthal Azo Iperoxo (PAI). a) PHX isomerization to the active *cis* isoform can be produced by 375 nm UV light and by 975 nm IR light in the presence of UV‐emitting UCNPs. b) Experimental setup to photoisomerize dark‐relaxed PHX in a vial containing UV‐emitting UCNPs under 975 nm IR illumination. c) The % *cis*‐PHX was quantified by aliquoting the vial solution at different IR illumination times and analyzing the *cis* and *trans* concentrations using HPLC. d) Time course of PHX activation with UCNPs under 975 nm light (red plot). The red and green boxes indicate the active and inactive PHX concentration ranges, respectively, based on the 1 µm concentration used in vitro for maximum photoswitching of activity.^[^ ^21^ ^]^ EC_50_ ∼ 5 nM. When a 2.5 mm thick pig skin sample is interposed (see Supporting Information), UV alone cannot photoswitch PHX (blue plot), whereas IR can photoswitch, albeit at a lower rate (pink plot). e) PAI isomerization to the active *trans* isoform can be produced by 460 nm light (after 365 nm light preirradiation) and by 975 nm IR light in the presence of 460 nm‐emitting UCNPs. f) Experimental setup to photoisomerize UV‐preirradiated PAI in a vial containing 460 nm‐emitting UCNPs under 975 nm IR illumination. g) The % *trans*‐PAI was quantified by aliquoting the vial solution at different IR illumination times and analyzing the *cis* and *trans* concentrations using UV–vis absorption spectroscopy. h) Time course of PAI activation with UCNPs under 975 nm light. The red and green boxes indicate the active and inactive PAI concentration ranges, respectively, based on the 200 nm concentration used in vitro for maximum muscarinic activation; EC_50_ ∼ 25 pM.^[^ ^35^ ^]^

Phototrexate, a photoisomerizable analog of the antifolate drug methotrexate in clinical use against cancer and psoriasis, was prepared as previously described by Matera et al.^[^ ^21^ ^]^ Proof of concept experiments of upconverted Phototrexate activation were carried out with water‐dispersed upconverting nanoparticles (UCNP, DiagNano Water Dispersible Upconverting Nanoparticles, catalogue no. DNL‐B006, Creative Diagnostics) emitting 365 nm light under 975 nm illumination. UV light was absorbed by Phototrexate and produced its isomerization from the trans inactive form to the cis active form, which acted as a competitive antagonist in the cellular enzyme dihydrofolate reductase (DHFR) with an IC_50_ = 6 nm.^[^ ^21^ ^]^ UCNPs were added to a standard HPLC glass vial containing dark‐relaxed (*trans*) Phototrexate, to give a final concentration of 20 µm Phototrexate and 5 mg mL^−1^ UCNPs. The mixture was illuminated with a continuous‐wave (CW) 980 nm collimated beam (2 mm diameter) from a laser diode (200 mW, model RLDH980‐200‐3, Roithner Lasertechnik GmbH, Austria) in a custom‐designed setup, equipped with a 1x Zoom lens and CMOS camera to visualize the laser beam and to ensure the beam position inside the vial. The setup was enclosed in a safety box to prevent reflections from the invisible laser (Class 3B). After each illumination time, aliquots were collected from to quantify the amount (%) of *cis* isomer by high performance liquid chromatography (HPLC) analysis using an XSelect C18 column (4.6 × 50 mm, 3.5 µm; Waters) with a mobile phase consisting of water/acetonitrile containing 0.1% formic acid (v/v), applied in a gradient from 5% to 100% acetonitrile over a 3‐min run (**Figure** **1**c). As a proof of concept to assess the validity of the method, the experiment was repeated using a 0.25 mm thick pig skin filter, irradiating at either 980 nm (laser) or 375 nm (LED) light.

#### 2.1.5 Photoisomerization of Muscarinic Ligand Phthalimide‐Azo‐Iperoxo (PAI)

PAI synthesis involves the preparation of the azobenzene spacer, as described by Riefolo et al.^[^ ^22^ ^]^, either a phthalimide allosteric fragment, or the final conjugation of the orthosteric Iperoxo‐like moiety. The molecular structure of PAI was confirmed through ^1^H NMR, ^13^C NMR, and mass spectrometry (MS), and its ability to modulate M_2_ muscarinic receptors was demonstrated in vitro and in vivo. The photoisomerization behavior of PAI was assessed via a UV–vis Spectrophotometer (Shimadzu V‐1800). The optimal wavelengths for *trans‐cis* and *cis‐trans* isomerization were 365 and 460 nm, respectively. *Trans‐*PAI acted as a dualsteric (orthosteric and allosteric) activator of M_2_ muscarinic receptors with an EC_50_ = 25 pM.^[^ ^22^ ^]^

Proof‐of‐concept experiments of upconverted PAI activation were carried out with water‐dispersed upconverting nanoparticles (UC: CaWO_4_ codoped with Er^3+^ and Yb^3+^) emitting 460 nm light under 975 nm illumination. This wavelength was absorbed by *trans*‐PAI and produced its isomerization from the *cis* inactive form to the *trans* form, which acted as a dualsteric activator of M_2_ muscarinic receptors.^[^ ^22^ ^]^ Green emitting UCs (CaWO_4_ codoped with Er^3+^ and Yb^3+^) were added to a cuvette containing 10 µm inactive PAI (*cis*‐PAI, obtained previously by illumination with 365 nm light during 5 min at 36 mW·cm^−2^) and illuminated with 980 nm light from a continuous wave (CW) laser source (Roithner LaserTechnick GmbH RLDH980‐200‐3 with 200 mW output power) in the same safety setup used for Phototrexate. After each illumination time, aliquots were collected from the cuvette to quantify the % of *cis* isomer using UV–vis absorption spectrometry.

Based on the results obtained with UCNPs in the solution, the ability of the scaffold was tested to influence the photoisomerization of PAI. A solution of *cis‐*PAI in a cuvette (pre‐illuminated with 365 nm light, during 5 min at 36 mW·cm^−2^) was placed in contact with the scaffold and exposed to a 980 nm laser (Roithner LaserTechnick GmbH RLDH980‐160‐3). Absorbance was measured over a 60‐min period to monitor changes caused by the interaction between PAI and the scaffold under near‐infrared (NIR) illumination. The scaffold, illuminated with UV light, contained PAI (10 µm) dissolved in Milli‐Q water and was subjected to 980 nm laser light to activate upconversion particles. PAI was positioned as a thin transparent film without direct contact with the scaffold and exposed to 980 nm light. Absorbance measurements were recorded at 20‐min intervals over a period of 1 h to observe changes due to the interaction between PAI and the scaffold under NIR illumination. Absorbance of PAI was measured regularly using a UV–vis spectrophotometer. The measurements were conducted under three distinct experimental conditions, including a dark condition (baseline absorbance): PAI in its trans (active) form was placed in a light‐free environment to assess its baseline absorbance characteristics. UV‐induced cis isomerization: PAI was exposed to UV light for 30 min, inducing the molecule into its inactive cis form. Absorbance was recorded to evaluate the molecular changes under UV irradiation. Interaction with scaffold under 980 nm light: PAI, in contact with the scaffold, was irradiated with 980 nm light, and absorbance was measured every 20 min over the course of 1 h. In a final step, the same conditions were repeated with the undoped X10 scaffold to determine if the light source alone influenced photoconversion.

### 2.2 In‐Vitro Dissolution

Scaffolds were immersed in Simulated Body Fluid (SBF) to assess their bio‐response and dissolution rate. The SBF was prepared according to Kokubo's Protocol as described in Ref. [32]. The SBF mimics the ionic concentration and pH of human plasma, making it ideal for assessing bioactivity.

Samples (n = 3) were immersed for different time points: 24, 72, and 336 h, in an incubator at 37 °C (±0.2 °C) and 120 rpm, to maintain a laminar flow. The sample mass to volume ratio was set to 10 mg mL^−1^. The pH of the immersion medium was measured with a pH‐meter (SevenMulti MP 225, Mettler Toledo International Inc., Greifensee, Switzerland) with an accuracy of ± 0.02. Furthermore, after each time point of immersion in SBF, the scaffolds were rinsed with acetone to stop the reaction, dried under a fume hood overnight. The solutions were analyzed by Inductively Coupled Plasma Optical Emission Spectroscopy (ICP‐OES 5100, Agilent Technologies, USA). 1 mL of the solution was collected from each container after each time point and diluted with 9 mL of 1 m HNO_3_. The examined elements were B (wavelength 349.772 nm), Ca (317.933 nm), Mg (280.270 nm), P (213.618 nm), Si (288.158 nm), Sr (421.552 nm), and W (220.449 nm).

The upconversion (UC) spectrum of the X10UC scaffold was measured prior to and after immersion in SBF using a TEC‐cooled fiber‐coupled multimode laser (II‐VI Laser Enterprise). The excitation was 980 nm, and the Spectro 320 optical spectrum analyzer (Instrument Systems Optische Messtechnik GmbH, Germany).

### 2.3 Cell Culture Study

#### 2.3.1 Isolation of hADSCs and Expansion

Human adipose stem cells (hADSCs) were isolated as described in Ref.[33] at the Tampere University Hospital, following the ethical approval of the Ethics Committee of the Expert Responsibility area of Tampere University Hospital (R15161). After isolation, these hADSCs were cultured in α‐Minimum Essential Media (α‐MEM) (Gibco, Life Technologies, Carlsbad, CA, USA) without nucleosides, with the addition of 1% penicillin/streptomycin and 5% human serum (Serana Europe, Germany GmbH). The cells were kept at 37 °C in a humidified incubator (Thermo Scientific Forma Steri‐Cycle i160 CO_2_) with 5% CO_2_ and 95% air environment until they reached 80% confluence.^[^ ^34^ ^]^

#### 2.3.2 Pre‐Incubation of Cells

The X10 and X10UC scaffolds, with an average diameter of 3.5 mm and an average height of 3.3 mm, were heat‐sterilized at 200 °C for 3h. The sterilized scaffolds were pre‐incubated for 24 h in 2 mL of 99% α‐MEM 1% P/S at 37 °C in an incubator with 15 RPM agitation, to suppress the risk of burst release of ions. Four scaffolds were used at each time point, with the positive control being the Tissue Culture Polystyrene (TCPS) 48‐well plate seeded with cells but without scaffolds. After each time point, the culture media were collected for ICP analysis. The solution was diluted 10 times in ultrapure water, and three samples per time point were analyzed along with a blank. All ICP values are presented as mean ±  standard deviation (SD).

#### 2.3.3 Live/Dead Assay

To assess the cytocompatibility of scaffolds in direct contact with cells, a live/dead assay was performed. Pre‐incubated scaffolds were placed in 48‐well plates (Thermo Fisher Scientific, Waltham, MA, USA). 25,000 hADCs were seeded in each well with 1 mL of αMEM. The tested time points (n = 3) were 24, 72, and 168 h. The culture medium was refreshed at 48 and 144 h. After each time point, the culture medium was collected for analysis using ICP‐OES.

The samples were rinsed with Dulbecco's Phosphate Buffered Saline (DPBS from Gibco, Life Technologies, Carlsbad, CA, USA). A staining solution, prepared according to the instructions of the manufacturer of the Live & Dead Kit (Invitrogen, Life Technologies, Carlsbad, CA, USA), was added to the wells and incubated at room temperature for 30 min. The staining solution consists of 1% (v/v) Calcein AM and 0.5% (v/v) Ethidium homodimer‐1, used to stain viable and necrotic hADSCs cells. Finally, the samples were rinsed with DPBS, and the cells were examined using a fluorescence microscope Olympus IX51 (Olympus Corporation, Japan).

#### 2.3.4 Cell Proliferation

To compare the viability of hADSCs in contact with the various types of scaffolds, the CyQUANT assay was performed by using the CyQUANT cell proliferation assay kit (Invitrogen, Life Technologies, Carlsbad, CA, USA). After seeding the hADSCs, the culture medium was refreshed at the respective time point (e,g, 48 and 144 h). After each time point, cells were lysed with 500µL 0.1% Triton X‐100 (Sigma–Aldrich, St Louis, MO, USA) and sorted at −80 °C. After one freeze‐thaw cycle, three 20 µL samples of each lysate were transferred to a wellplate and mixed with 180 µL of CyQUANT GR dye solution. An Elmer Spectrofluorometer (VICTOR Nivo Multimode) was used to measure the fluorescence at 520 nm. The statistical analysis software utilized was GraphPad Prism 8.

#### 2.3.5 Morphology

The cell morphology was investigated on days 1, 3, and 7. A total of 25 000 hADSCs were cultivated in 1 mL of α‐MEM medium (containing 1% P/S, 5% human serum, and no glutamine) and deposited directly on top of the scaffolds. The culture medium was refreshed at 72 and 144 h. The control was 10 mm diameter glass coverslips (Marienfeld, Lauda‐Konigshofen, Germany) in a 48 wellplate.

At each time point, the cells were fixed with 4% (w/v) para‐formaldehyde solution in Phosphate Buffered Saline (PBS; AlfaAesar, Haverhill, MA, USA) for 15 min at each time point. Afterward, cells were permeabilized for 10 min with 0.1% (v/v) Triton X‐100 (Sigma–Aldrich, St Louis, MO, USA). The scaffolds (with cells) were incubated in PBS (Medicago AB, Uppsala, Sweden) containing 3% (w/v) Bovine Serum Albumin (BSA, Sigma–Aldrich, St Louis, MO, USA) for 45 min to block non‐specific binding sites.

The cell's cytoskeleton and nucleus were stained for 45 min using FITC‐labelled phalloidin (1:500) (Sigma–Aldrich, St Louis, MO, USA P1951) and 4’,6‐Diamidino‐2‐phenylindole dihydrochloride (1:2000) (DAPI, Sigma–Aldrich, St Louis, MO, USA D9542), respectively. Samples were rinsed with PBS–BSA 0.5% and pure water and imaged with a LSM800 confocal microscope (Zeiss, Jena, Germany).

## 3 Results and Discussion

### 3.1 Proof of Concept of Upconverted Activation of Photoswitchable Drugs Phototrexate and PAI

To test the feasibility of activating drugs with NIR light via the luminescence emitted by UCNPs, we carried out proof‐of‐concept experiments by adding to a HPLC glass vial previously reported photoswitchable drugs and commercial UCNPs with a suitable emission wavelength. In particular, we combined Phototrexate (a photoswitchable antiproliferative compound derived from the antifolate drug methotrexate) with [NaYREF_4_, RE:Yb, Er, Tm, Gd, Mn, Lu] UCNPs emitting 365 nm UV light, and Phthal Azo Iperoxo (PAI, a photoswitchable dualsteric activator of M_2_ muscarinic acetylcholine receptors) with CaWO_4_ codoped with Er^3+^ and Yb^3+^ UCs, emitting green light. The structures of Phototrexate and PAI, and their optimal wavelengths of isomerization, are shown in Figure 1a,e. The experimental schematic is presented in Figure 1b,f. The time course of drug activation under NIR light is shown in Figure 1c,d for phototrexate and g and h for PAI, and indicates steady photoisomerization in the presence of the corresponding UCNPs. Importantly, the concentrations used in both experiments allow measuring the absorbance and photo‐isomerizing of the compounds to a degree that surpasses the reported in vitro potency (IC_50_ and EC_50_ concentrations of Phototrexate and PAI, respectively), and in the case of Phototrexate, it allows quantifying the actual % *cis* isomer using HPLC. Thus, the UCNPs demonstrate an ability to photoactivate the drugs in a pharmacologically relevant range. To further evaluate the potential of UCNP‐mediated drug activation under biologically relevant conditions, we repeated the Phototrexate experiment using a pig skin sample as an optical filter between the NIR source and the sample. Despite the expected attenuation of upconverted emission due to tissue absorption, we still observed a robust and encouraging degree of photoconversion upon 975 nm irradiation. Remarkably, this conversion was significantly higher than that obtained with direct 375 nm irradiation through the same pig skin filter, which was unable to induce substantial isomerization. These results underscored the ability of UCNPs to generate sufficient luminescence for drug activation even in the presence of biological barriers.

These promising results prompted further advances in our upconverted photoactivation methodology, by embedding the luminescent particles in a solid scaffold that can be easily manipulated, that embeds the potentially toxic nanoparticles and prevents their release to the medium, and that allows for conceptualizing the resulting material as a potentially implantable device.

### 3.2 In‐Vitro Dissolution ICP of X10 and X10UC Prior to Cell Test

While for the proof‐of‐concept, UCNP (NaYF_4_) was used, studies have shown that not only this UC crystals tend to degrade in aqueous solution, but they also degrade upon glass sintering.^[^ ^36^ ^]^ Therefore, scaffolds were processed with CaWO_4_ crystals, known to have comparable efficiency and to be more stable in solution.^[^ ^26^ ^]^ The physicochemical properties of the 3D printed scaffolds X10 and X10UC were investigated and are presented in the Section S1 (Supporting Information). Furthermore, an up‐conversion intensity of 5.19 ± 0.20 (in 10^−7^ Wcm^−2^) was obtained under 980 nm excitation at 4.2 Wcm^−2^ power density. These indicate ≈10^−5^% up‐conversion efficiency per Wcm^−2^ of excitation. According to,^[^ ^37^ ^]^ the same value for the green up‐conversion of the archetypical NaYF_4_:Yb,Er phosphor is ≈5% per Wcm^−2^ at 200 Wcm^−2^ excitation irradiance. This suggests rather good efficiency for our materials when taken into account that the concentration of the UPC particles in our materials is 10 wt% and that our setup has a much lower excitation irradiance.

**Figure 2:**
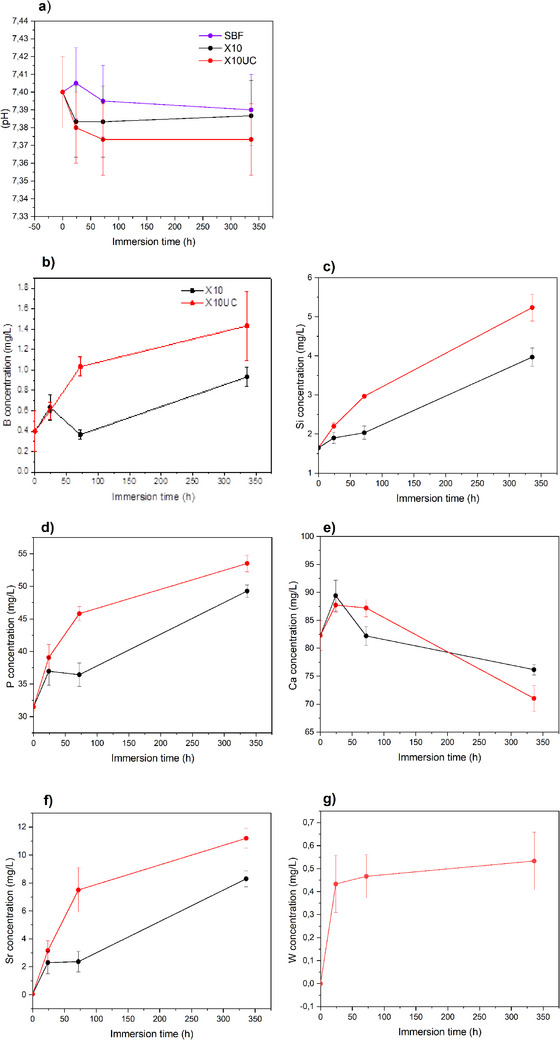
The pH (a) of solutions and concentration of B (b), Si (c), P (d), Ca (e), Sr (f), W (g) in the SBF.

Scaffolds were immersed in SBF for up to 2 weeks (336 h), and the pH of the SBF was recorded at each time point (**Figure** **2**a). During the immersion period, the pH of the immersion solutions remained stable within the accuracy of the measurements and irrespective of the scaffolds. Typically, a slight decrease in pH is expected when immersing metaphosphate glasses due to the release of phosphorus in the solution. Indeed, in Ref.[31], a small decrease in pH for the glass X10 was reported; however, in Ref.[31], X10 was studied in powder form, whereas in this study, scaffolds, having a smaller surface area compared to particles, were tested. Therefore, when compared to the powder sample, a slower dissolution rate can be anticipated in the case of scaffolding, thus reducing the pH changes.

Following the in vitro dissolution test, the ion release was recorded using ICP‐OES. Figure 2b–g present the boron (B), silicon (Si), phosphorus(P), calcium (Ca), strontium (Sr), and tungsten (W) concentration, in SBF solution, as a function of immersion time. B (b), Si(c), P(d), and Sr(e) concentrations increase in the SBF with increasing immersion time. The increase appears to slow down between 72 and 168 h of being immersed. At all‐time points, the release of those ions appears to be greater in the case of the X10UC compared to X10.

**Figure 3:**
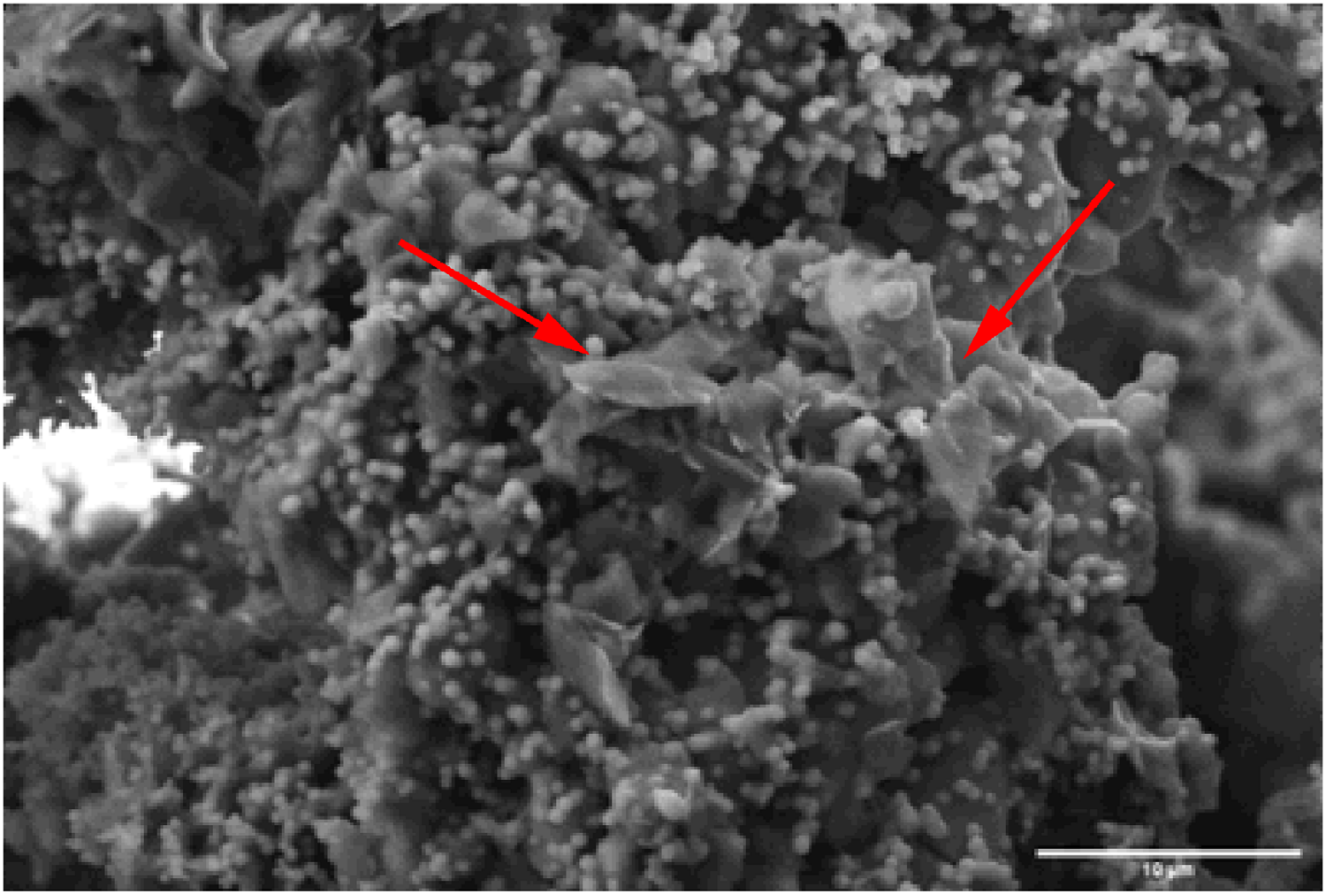
The reactive DCPD layer's morphology shows a mixture of platelet and nodule structures, scale bar:10 µm.

The increased ion release (B, P, Si, Sr) over time is assigned to the dissolution of the phosphate glass in SBF. A slightly higher ion concentration can be seen for the UC‐containing scaffolds, and it is likely due to the presence of UC crystals, which may partially interfere with the sintering process. While the overall scaffolds porosity (69 + (2.5)%) remains unchanged, regardless of the presence or not of UC crystals, it is well known that the addition of such particle, with a softening temperature much higher than the sintering temperature used in this study, leads to defect within the struts, thus slightly increasing the overall surface area.^[^ ^38^, ^39^ ^]^ With an increase in specific surface area. This would inevitably lead to a slightly increased dissolution rate. The concentration of Ca, as opposed to the previously mentioned ions, increases during the first 24 h and, for longer immersion times, decreases. A decrease in Ca concentration is linked to its consumption caused by the precipitation of a reactive layer. While the reactive layer was found, in Ref.[31], to be a dicalcium phosphate dihydrate (DCPD), which generally has a plate‐like structure, here a mixture of platelets and nodules was seen (**Figure** **3**). Santos Vilela et al. reported that at pH 6.5, the precipitation of DCPD is favored in highly saturated solutions, whereas at higher pH, low crystallinity HAp is predominantly precipitated.^[^ ^40^ ^]^ Here, it is conceivable that a mixture of low crystallinity‐Hap and DCPD was formed during the bioactivity test.^[^ ^41^ ^]^ The consumption of calcium ions, to form a CaP reactive layer, also coincides with the decrease in the rate of release of B, Si, P, and Sr ions. A decrease in the dissolution rate can be expected when a reactive layer precipitates at the glass surface.^[^ ^31^ ^]^ The reactive layer precipitation, as seen by the decrease in Ca, does not appear to be significantly impacted by the presence of the UC crystals.

It is noteworthy that W is found in the SBF solution, the concentration of which increases over time, suggesting a slight release of the UC crystals upon immersion in SBF. While studies on tungsten carbide (WC) have shown that W ions do not present a significant toxicity, as opposed to WC particles,^[^ ^42^ ^]^ it is of paramount importance to demonstrate that, in our case, the release of W does not have a negative effect on cell viability and proliferation.

### 3.3 Cytocompatibility Test

**Figure 4:**
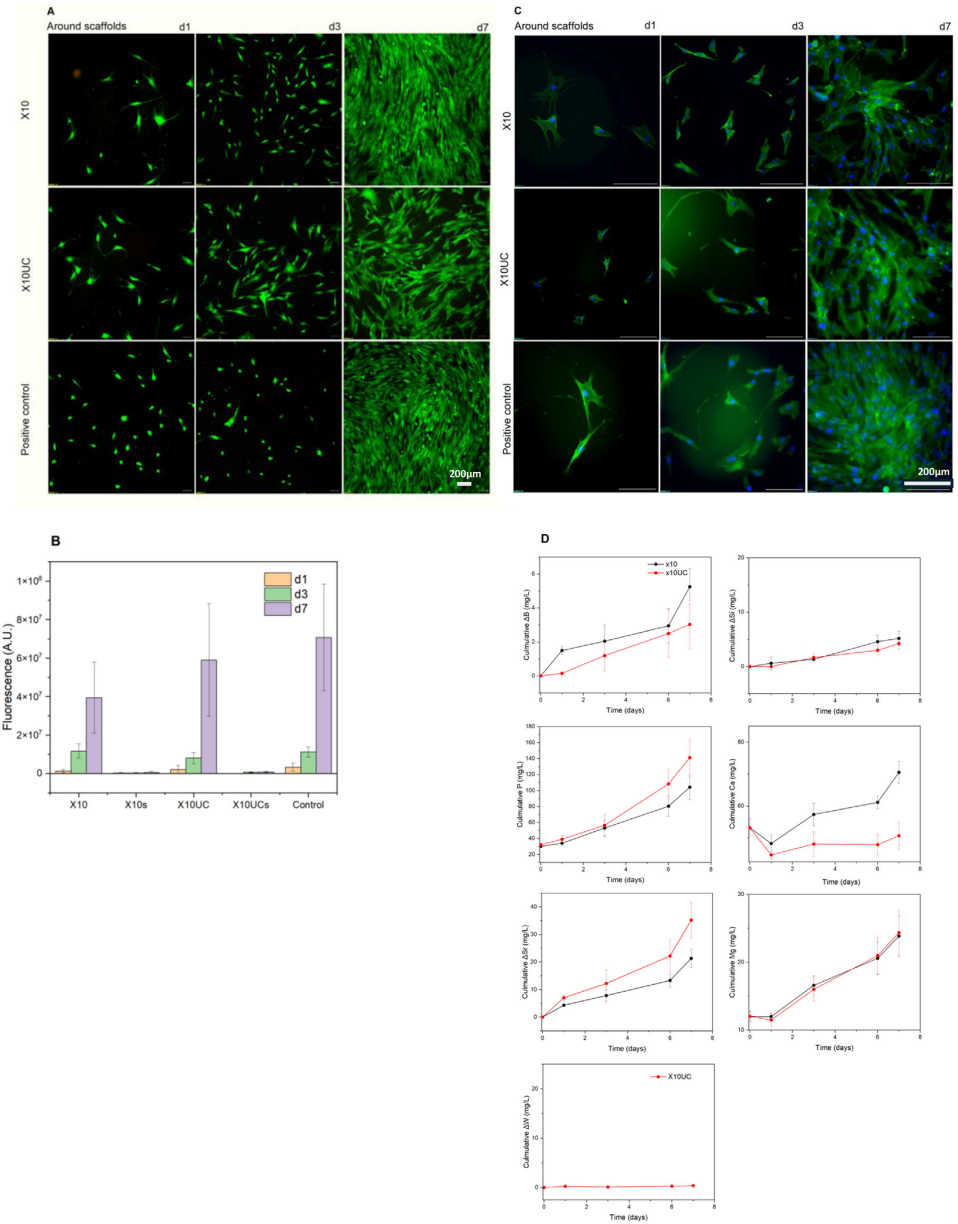
A) Preliminary cell study for X10 and X10UC scaffolds. hADSCs seeded at 25 000 cells cm^−2^ in 1 mL αMEM per well. Scaffolds were pre‐incubated for 1 day in 2 mL αMEM. Green fluorescence indicates viable cells, and red fluorescence shows necrotic cells, stained with Calcein AM and Ethidium homodimer‐1, respectively. Scale bar: 200 µm. B) Proliferation of hADSCs cultured with X10 and X10UC scaffolds in αMEM for 1, 3, and 7 days, assessed using the CyQUANT assay. X10 and X10UC represent cells in the solution collected from around the scaffolds, while X10s and X10UCs correspond to cells attached to the top of the respective scaffolds. C) Morphology of hADSCs analyzed by cytochemical staining of nuclei (DAPI, blue) and cytoskeleton (phalloidin, green) after 1, 3, and 7 days of culture in αMEM. Scale bar: 200 µm. D) Cumulative ion concentrations from ICP analysis on days 1, 3, and 7 of cell proliferation. n = 3 scaffolds per condition.

The impact of the X10 and X10UC scaffolds on hADSCs viability, proliferation, and morphology was investigated in direct contact. Scaffolds were pre‐incubated for 1 day, and the hADSCs viability was assessed by fluorescence microscopy at 1, 3, and 7 days of culture. **Figure** **4**A shows the viable cells (in green) around the scaffolds. The cells appear to spread at the bottom of the wellplate, leading to a spindle‐like shape, as expected from this cell line. Over the 7 days of the culture period, the cells are proliferating around scaffolds. Proliferation was assessed using the CyQUANT assay, presented in Figure 4B. The wellplate, without scaffolds, was used as a control. The proliferation of the cells, around the scaffolds and in the control, increases exponentially until day 7. Such proliferation follows a similar trend already presented for this cell line in Ref. [43]. It is noteworthy that the cell density is statistically similar regardless of the condition at each time point. The ions released from both scaffolds, and potentially from the CaWO_4_ crystals, did not affect cell growth and proliferation.

The cells’ viability and proliferation were also studied on top of scaffolds. The cells' attachment to the surface of the glass was low (not presented). Cell proliferation was studied by CyQUANT^TM^ assay (Figure 4B). Cells were quantified from the solution collected from around the X10 and X10UC scaffolds and in contact with cells. The proliferation curve, presented in Figure 4B, also confirms the low attachment ability of cells and limited proliferation at the surface of scaffolds. This is most likely due to the congruent dissolution of phosphate glasses.^[^ ^28^ ^]^ Such phosphate glasses can be considered bioresorbable, as cells are keener to proliferate around the scaffolds, rather than at the surface.^[^ ^44^ ^]^

Cell morphology (Figure 4C) provides insights into the cell's behavior over the 7‐day culture period. On day 1, the cells exhibit distinct nuclei and elongated actin filaments. These cellular extensions suggest early attachment and interaction with the surface of the wellplate, as actin plays a critical biological role in sensing the extracellular environment and promoting adhesion. The distinct nuclei indicate that the cells are healthy and actively engaging with their surroundings, setting the stage for progressive cellular activities. By day 3, active cell proliferation and migration were observed, reflecting a positive response to the culture condition. On day 7, in all conditions, a cell matt was imaged in all conditions.

While ion release was studied in SBF, SBF testing does not fully mimic the immersion condition during the cell culture test.^[^ ^30^ ^]^ Therefore, ion release in the culture medium was also assessed to better understand the changes in ion concentration experienced by the cell. Figure 4D, presents the B, Si, P, Ca, Sr, Mg, and W. As opposed to dissolution in SBF where the X10UC scaffold appeared to dissolve faster (Figure 2), no significant differences in ion release were observed between the two scaffolds in culture medium. Furthermore, as expected from surface reacting material, the ion release in culture medium is faster than in SBF, due to the lower volume of solutions used in such tests. From Figure 4D, ions from both scaffolds are steadily released over the 7 days of culture. Despite the significant release of phosphorus, no adverse cell behavior could be evidenced, indicating that the release of all ions is beneficial to the cell activity. It is noteworthy that while tungsten was clearly released into the SBF (Figure 4D), no tungsten could be measured during cell culture. In SBF, it appears that tungsten was released within the first 24 h, and, for longer immersion times, the tungsten concentration remains constant. It is therefore likely that all soluble tungsten was released during the pre‐incubation prior to the cell culture after 24 h.

### 3.4 Upconversion Spectra of the Scaffolds Prior to and after Immersion in SBF (λ_exc_ = 980 nm)

**Figure 5:**
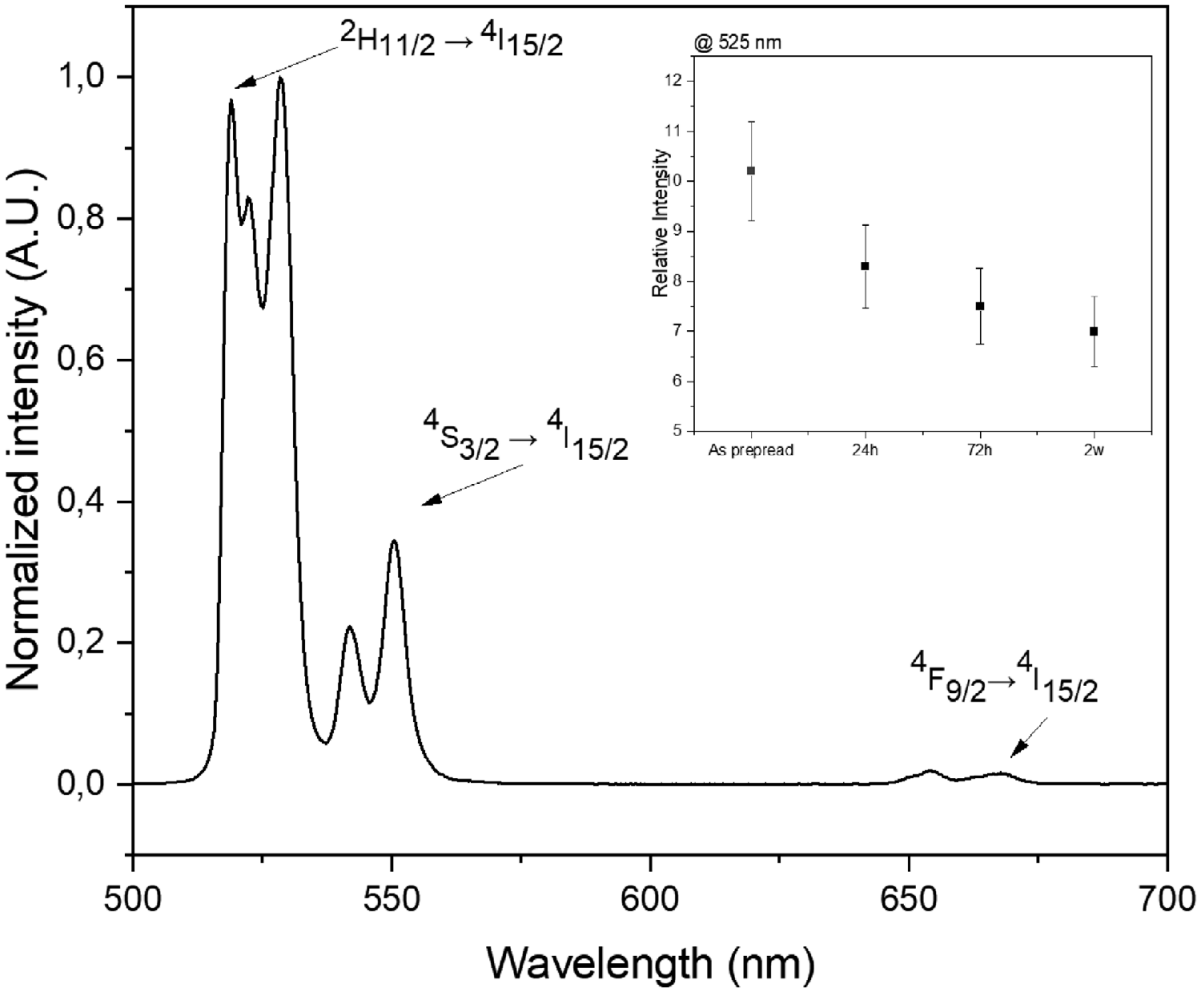
Normalized upconversion spectra of the scaffolds under excitation at 980 nm. As the shape of the recorded bands remains unchanged for the as‐prepared sample and after immersion in SBF for 24 h, 72 h, and 2 weeks, the relative intensity @525 nm of scaffolds is displayed in the inset.

The upconversion spectra of the scaffolds were recorded prior to and after immersion in SBF for 24h, 72h, and 336h, using an excitation wavelength of 980 nm. As shown in **Figure** **5**, the recorded spectra exhibit emission bands at 525 and 550 nm as well as at 650 nm. These bands are typical emission bands from Er^3+^ and correspond to ^2^H_11/2_ → ^4^I_15/2_, ^4^S_3/2_ → ^4^I_15/2_and ^4^F_9/2_→^4^I_15/2_ transitions of Er^3+^, respectively. Although no changes in the shape of the emission bands were observed after immersion in SBF, the intensity of the emission bands progressively decreases overtime (see inset of Figure 5) confirming that 1) the UC crystals are released progressively from the scaffolds to the medium and 2) the immersion in SBF has no impact on the local environment of Er^3+^ in the UC crystals which remains unchanged. It is clear from Figure 5 that the scaffolds still emit green light after at least 2 weeks of immersion in SBF.

### 3.5 Nitric Oxide Release

**Figure 6:**
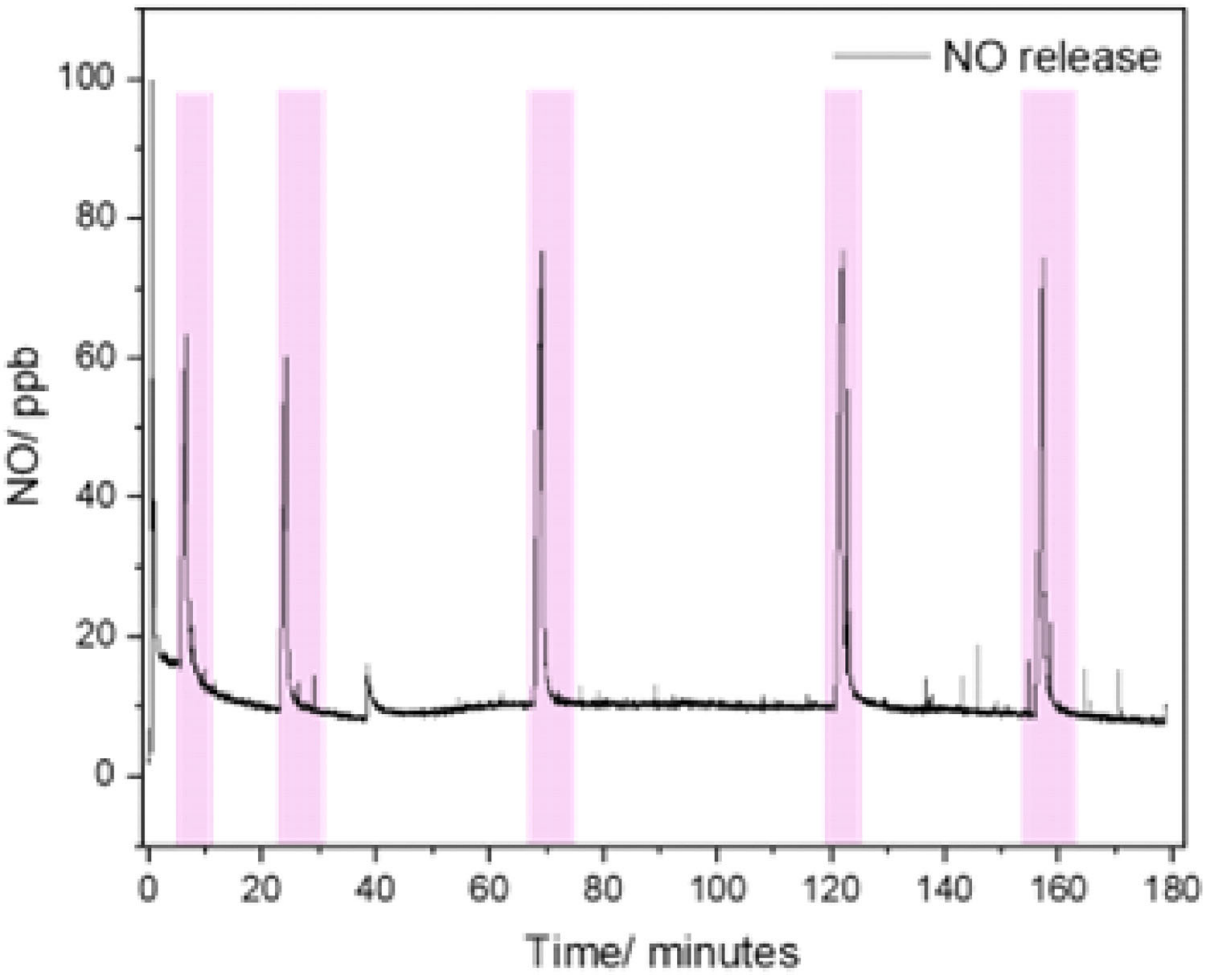
Nitric oxide (NO) release over 180 min (λ_exc_ = 980 nm). NO release over 180 min under 980 nm laser, with peaks corresponding to laser activation. Peaks indicate NO release during laser‐on periods.

The scaffolds were covered with SNAP and irradiated with a 980 nm laser as in Ref. [26]. **Figure** **6** presents the NO concentration release from the scaffold as a function of time. The NO release clearly aligns with the activation of the laser, and the NO release rapidly drops when the laser is switched off, confirming that the green UC from the scaffold is strong enough. The reproducibility of the peaks suggests a consistent response mechanism of NO release under 980 nm laser stimulation. As in Ref.[26], the irradiation at 980 nm alone cannot lead to NO photoactivation (not shown here).

### 3.6 Absorbance Spectra and Cis‐to‐Trans Isomerization Kinetics of PAI Under Different Light Conditions

The photoswitchable muscarinic modulator PAI (Figure 1) was isomerized to the *trans* active form (M_1_ mAChR agonist) using light emitted from the UC scaffold. The scaffold contains UCNP, which absorbs 980 nm near‐infrared (NIR) light and emits green light, enabling indirect activation of PAI, which is optimally photoisomerized at 460 nm. This system allows precise control over *trans‐cis* isomerization via optically induced switching.

**Figure 7:**
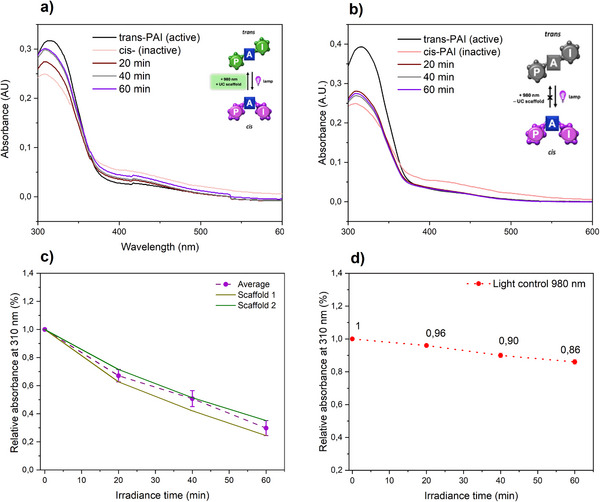
Upconverted photoisomerization of the muscarinic drug PAI using UC scaffolds and IR light. UV–vis absorbance spectra of 10 µm PAI in MilliQ water under 980 nm irradiation with (a) and without (b) UC scaffolds. c) Relative absorbance of PAI with different scaffolds, showing a time‐dependent decrease. d) without UCNP scaffold as light controls.

**Figure** **7** presents the absorbance spectra of 10 µm PAI in Milli‐Q water under different irradiation conditions. The primary absorption band at 310 ± 3 nm corresponds to the *π*–*π* ^*^ transition of the *trans*‐isomer of PAI. The relative absorbance (Figure 7c,d) was calculated from the amplitude of the absorbance peak at 310 ± 3 nm (Figure 7a,b) is proportional to the concentration of *trans‐*PAI (active isomer). It reaches a minimum after UV preillumination, which converts PAI to the *cis* isomer (Figure 1g). We measured this absorbance peak as a function of 980 nm irradiation time in the presence of the UCNP scaffold, from the minimum value (after UV) up to 60 min (Figure 7c,d). This measurement reports of the upconverted photoisomerization rate (Figure 7c) and can be compared to the slower thermal relaxation that occurs under IR irradiation in the absence of UCNP scaffolds (Figure 7d). We calculated the relative absorbance (%) as a function of time using the equation below:

(1) $$A_{relative}=\frac{A_{\lambda }\left(dark\right)-A_{t}}{A_{\lambda }\left(dark\right)-A_{\lambda }\left(UV\right)}*100$$

where *A*_λ_ (dark) is the absorbance measured in the absence of UV light (baseline or initial state), A_t_ is the absorbance at a given time t, and *A*_λ_ (UV) is the absorbance after full UV exposure.

The results (Figure 7c,d) show the change in absorbance peak between the initial state and each time due to IR irradiation. This kinetic analysis reveals a time‐dependent decrease in *cis*‐isomer concentration and then its conversion to the *trans*‐isomer upon exposure to 980 nm light in the presence of the UC scaffold.

PAI displays a strong absorbance signal at 311 ± 3 nm that is stable (*trans*) in the absence of illumination. Upon UV light exposure for 30 min, PAI photoisomerizes to its *cis* form, leading to a decrease in absorbance at this wavelength and a reduction in pharmacological activity. In dark conditions, *cis‐*PAI relaxes thermally in the range of hours, so in the 1 h time range of our experiments, it is stable in the *cis* state (Figure 7d). Under 980 nm irradiation in the presence of the UC scaffold, PAI isomerizes to the *trans* state (active isoform), confirming the role of UC‐mediated green light emission in triggering photoisomerization. The absorbance change upon UV exposure confirms *cis*‐isomer formation, while its reversal under 980 nm irradiation (via UC‐generated green light) indicates a return to the *trans* state. These results validate the effectiveness of green‐light‐induced photoisomerization via upconversion crystals, demonstrating a controlled and reversible molecular switching mechanism.

Importantly, when the 980 nm laser was used without the UC scaffold, no significant *trans*‐isomer formation was detected (n = 2), proving that green UC emission is essential to trigger the isomerization process.

These findings demonstrate that PAI undergoes efficient photoisomerization by UC‐mediated green light emission, enabling a controlled transition between *cis* and *trans* states. The presence of the UC scaffold plays a crucial role in the isomerization process, distinguishing it from direct UV or NIR irradiation alone. However, it should be pointed out that the isomerization is rather slow and not yet optimal for biological application. Improving the UC efficiency is crucial to photoisomerize the drug at shorter irradiation times.

## 4 Conclusion

Bioresorbable scaffolds with embedded Er^3+^, Yb^3+^ co‐doped CaWO_4_ crystals with interconnected porosity were successfully 3D‐printed and characterized. The in vitro dissolution studies were conducted over two weeks and confirmed that the glass scaffolds degrade congruently and support the formation of a mix of DCPD/low crystallinity HA precipitation. The addition of the Er^3+^, Yb^3+^ co‐doped CaWO_4_ crystals in the scaffolds did not interfere with the ion release pattern. Due to the release of CaWO_4_ into the solution, the intensity of the green and red emission under 980 nm pumping from the scaffolds slightly decreases upon immersion in SBF over time. Cell studies confirmed excellent biocompatibility of the biophotonic scaffold with hADSCs. The controlled release of ions from the scaffolds, including boron, silicon, calcium, and strontium, supported cellular proliferation and activity. Importantly, the minimal tungsten release from CaWO_4_ particles did not adversely affect cell viability or proliferation, ensuring the materials’ safety and nontoxicity in in vitro studies.

The scaffolds were functionalized with SNAP and demonstrated NO release over 180 min under 980 nm pumping. In addition, the scaffolds exhibited an effective photoswitching response under NIR 980 nm laser irradiation, allowing precise temporal control over therapeutic agent release (PAI). Notably, this photoactivated release could be maintained up to 60 min after laser stimulation, showing the scaffolds’ potential for light‐triggered drug delivery.

These findings offer a promising platform for advanced drug delivery systems. The combination of controlled ion release, biocompatibility, and tunable photoactivated response underscores their versatility for therapeutic applications, particularly for precise drug release and regenerative properties, and for localized, on‐demand activation of drugs. Furthermore, the demonstrated bio‐response, stability, and ability to sustain cellular activity make these scaffolds promising candidates for tissue repair and regeneration.

This UC‐assisted mechanism has potential applications in photoresponsive biomaterials, controlled drug release, and optically controlled molecular switches in materials and phototherapies. Future studies should explore optimization of UC particle composition, brightness, spectral distribution, increasing isomerization efficiency, and potential applications in biological environments.

## Conflict of Interest

The authors declare no conflict of interest.

## Supporting information



Supporting Information

## Data Availability

The data that support the findings of this study are available from the corresponding author upon reasonable request.
