# Supplementary material for: Novel 3D‐Printed Biophotonic Scaffold Displaying Luminescence under Near‐Infrared Light for Photopharmacological Activation and Biological Signaling Compound Release
Source: Adv Healthc Mater. 2025 Aug 18;15(8):e02163. doi: 10.1002/adhm.202502163 (PMC12927531; doi:10.1002/adhm.202502163)
Supplement: Supplementary file 1 — Supporting Information [file ADHM-15-0-s001.docx]

Supplementary data

**Novel 3D printed biophotonic scaffold displaying luminescence under near-infrared light for photopharmacological activation and biological signaling compound release**

S. Ghanavati,^1,11+^ E. Opar,^2,3,4,+^ V.A. Gobbo, ^1^ C. Matera,^2,5^ F. Riefolo,^2,6^ R. Castagna,^2,7,8^ J. Colombelli,^10^ A. Draganski,^9^ J. Baggott, ^12^ M. Lastusaari, ^12^ P. Gorostiza,^2,4,11,*^, L. Petit^11^, J. Massera^1*^

1. Faculty of Medicine and Health Technology, Tampere University, Korkeakoulunkatu 3, Tampere 33720, Finland
2. Institute for Bioengineering of Catalonia (IBEC), The Barcelona Institute for Science and Technology (BIST), Carrer de Baldiri Reixac 10, Barcelona, 08028 Spain
3. Universitat de Barcelona Graduate Program.
4. Network Biomedical Research Center in Biomaterials, Bioengineering, and Nanomedicine (CIBER-bbn), Barcelona, Spain
5. Department of Pharmaceutical Sciences, University of Milan, Via L. Mangiagalli 25, 20133 Milan, Italy.
6. Teamit Institute, Partnerships, Barcelona Health Hub, Barcelona 08025, Spain
7. Dipartimento di Chimica, Materiali e Ingegneria Chimica Giulio Natta, Politecnico di Milano, Piazza Leonardo da Vinci 32, Milano, 20133 Italy
8. Department of Biotechnology, Latvian Institute of Organic Synthesis, Aizkraukles 21, Riga, 1006, Latvia Institute for Biomedical Research of Barcelona (IRBB), The Barcelona Institute for Science and Technology (BIST), Barcelona, Spain
9. Zylö Therapeutics, Greenville, SC, USA.
10. Catalan Institution for Research and Advanced Studies (ICREA), Barcelona, Spain.
11. Photonics Laboratory, Tampere University, Korkeakoulunkatu 3, Tampere 33720, Finland
12. Department of Chemistry, University of Turku, FI-20014 Turku, Finland

* Correspondence: jonathan.massera@tuni.fi (J. M.), [pau@icrea.cat](mailto:pau@icrea.cat) (P. G.)

1. Mechanical properties of scaffolds

Post processing the scaffolds were tested for their mechanical properties. As shown in Table S1, the Young’s modulus and fracture strength are comparable to values reported for cancellous bone [1]. Notably, the addition of upconverter crystals did not adversely affect the mechanical properties of scaffolds.

Table S1 . Mechanical properties, in compression of X10 and X10UC.

| Sample | Young modulus (MPa) | Fracture strength (MPa) |
| --- | --- | --- |
| X10 | 590 ± 130 | 17 ± 4 |
| X10UC | 450 ± 25 | 15 ± 4 |

1.2 Crystal structure of CawO_4_

The crystal structure of the upconverters was measured post-processing (Figure S1 a). The produced Er/Yb doped crystals exhibit diffraction patterns in agreement with CaWO_4_ crystals (ICDD card No. 98-006-0548). The normalized upconversion emission of the UC crystals under 980 nm laser (Fig. S1 b), exhibits the characteristic green and red emissions of Er^3+^ ions. Specifically, Fig. 1 b shows, under 980 nm excitation, the green emission corresponding to the transitions from the ^2^H_11/2_ and ^4^S_3/2_ levels to the ^4^I_15/2_ ground state, while the red emission arises from the ^4^F_9/2_→^4^I_15/2_ transition, consistent with typical Er³⁺ upconversion behavior.

The incorporation of the crystals in the scaffolds does not change the crystals structure of the upconverter (Figure S1 a). As shown in the manuscript the luminescence properties of the crystals are maintained post scaffold processing.

Figure S1. XRD pattern of the scaffold prepared with 10 wt% of 15Yb-0.75Er CaWO_4_ crystals compared to the synthesized 15Yb-0.75Er CaWO_4,_ shown together with reference diffraction lines for CaWO₄ from ICDD card No. 98-006-0548. (a). Normalized upconversion spectrum of the 15Yb – 0.75Er crystal (λ_exc_ = 980 nm) (b)

1.3 Compositional analysis

Figure S2 presents the SEM/EDX mapping for the X10UC scaffold. The SEM image (Fig. S2 a) shows a dense and well-sintered structure, where crystalline regions are distinctly visible and appear well embedded within the surrounding glass matrix. These regions exhibit good interfacial contact with the amorphous phase, suggesting effective incorporation during the synthesis process.

Elemental mapping was performed to confirm the distribution of key elements within the sample. The maps for phosphorus (P) (Fig. S2 b), calcium (Ca) (Fig. S2 c), and tungsten (W) (Fig. S2 d) show the spatial distribution of each element. Notably, the bright areas in the W map indicate tungsten-rich part, confirming the presence of CaWO₄ crystals embedded within the glass matrix. In contrast, phosphorus is more uniformly distributed, as it is primarily associated with the glass matrix. The Ca map (Fig. S2 c) also shows widespread distribution across the sample, which is expected, since calcium is present in both the glass matrix and in the CaWO₄ crystals.

The sharp contrast in the elemental maps of Figs. S2 b and S2 d, along with the absence of elemental diffusion into the surrounding matrix, suggest that the crystals are chemically stable and remain well confined within the glass host, without significant interfacial reaction or degradation


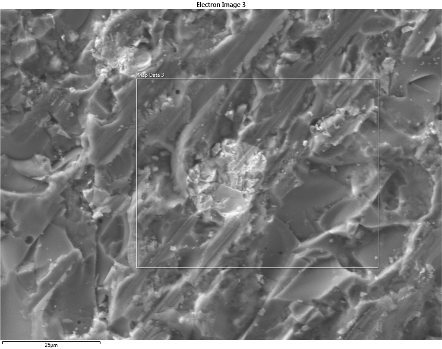


**a)**


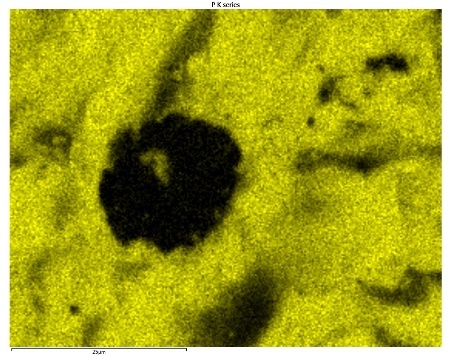


**b)**


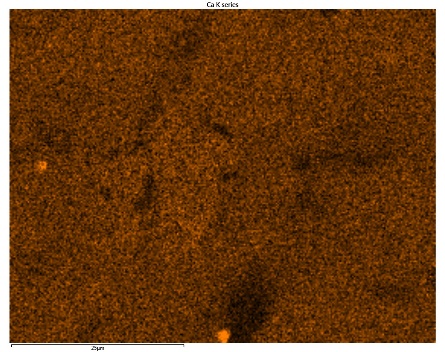


**c)**


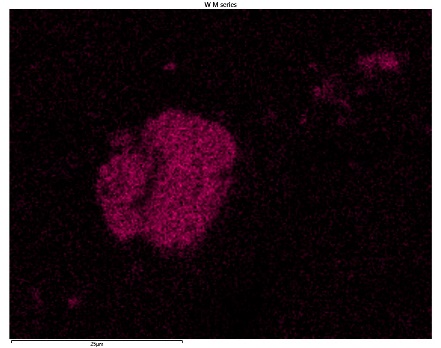


**d)**

Figure S2. SEM image of the X10UC sample. (a) Elemental maps of phosphorus (P), calcium (Ca), and tungsten (W), respectively (Fig. 2 b-d). Scale bar: 25 µm)

References

[1] E. B. W. Giesen, M. Ding, M. Dalstra, and T. M. G. J. van Eijden, "Mechanical properties of cancellous bone in the human mandibular condyle are anisotropic," Journal of Biomechanics, vol. 34, no. 6, pp. 799–803, 2001. <https://doi.org/10.1016/S0021-9290(01)00030-6>.
